# NaSr(AsO_4_)(H_2_O)_9_: the (Sr,As) analogue of nabaphite and nastrophite

**DOI:** 10.1107/S1600536809040355

**Published:** 2009-10-10

**Authors:** Matthias Weil

**Affiliations:** a Institute for Chemical Technologies and Analytics, Division of Structural Chemistry, Vienna University of Technology, Getreidemarkt 9/164-SC, A-1060 Vienna, Austria

## Abstract

The crystal structure of the title compound, sodium strontium orthoarsenate(V) nona­hydrate, is isotypic with NaSr(PO_4_)(H_2_O)_9_ and the minerals nabaphite [NaBa(PO_4_)(H_2_O)_9_] and nastrophite [Na(Sr,Ba)(PO_4_)(H_2_O)_9_]. The Na and Sr atoms are located on threefold rotation axes and are in the centres of slightly distorted Na(H_2_O)_6_ octa­hedra and Sr(H_2_O)_9_ tricapped trigonal prisms, respectively. A framework structure is established *via* edge-sharing of these polyhedra. Disordered AsO_4_ tetra­hedra (with threefold symmetry) are situated in the inter­stitial space of the framework. Although reasonable H-atom positions of the water mol­ecules were not established, close O⋯O contacts between the disordered AsO_4_ tetra­hedra and the water mol­ecules suggest strong O—H⋯O hydrogen bonding.

## Related literature

For a previous study of the title compound that revealed cubic symmetry and the lattice parameters, see: Ariguib-Kbir & Guerin (1973 ▶). Isotypic structures have been reported for synthetic NaSr(PO_4_)(H_2_O)_9_ (Takagi *et al.*, 1982 ▶), nabaphite [NaBa(PO_4_)(H_2_O)_9_] (Baturin *et al.*, 1982 ▶) and nastrophite [Na(Sr,Ba)(PO_4_)(H_2_O)_9_] (Baturin *et al.*, 1981 ▶). For crystal structures in the Sr—As—O—(H) system, see: Mihajlovic & Effenberger (2006 ▶); Weil *et al.* (2009 ▶). As—O bond-length data for tetra­hedrally coordinated arsenic were compiled and computed by Baur (1981 ▶) and Schwendtner (2008 ▶). For ionic radii, see: Shannon (1976 ▶).

## Experimental

### 

#### Crystal data

NaSr(AsO_4_)(H_2_O)_9_
*M* *_r_* = 411.67
Cubic, 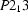

*a* = 10.6435 (1) Å
*V* = 1205.74 (2) Å^3^
*Z* = 4
Mo *K*α radiation
μ = 7.29 mm^−1^
*T* = 296 K
0.36 × 0.24 × 0.24 mm

#### Data collection

Bruker APEXII CCD diffractometer
Absorption correction: multi-scan (*SADABS*; Bruker, 2008 ▶) *T*_min_ = 0.179, *T*_max_ = 0.274
41355 measured reflections
3435 independent reflections
3272 reflections with *I* > 2σ(*I*)
*R*_int_ = 0.037

#### Refinement

*R*[*F* ^2^ > 2σ(*F* ^2^)] = 0.023
*wR*(*F* ^2^) = 0.056
*S* = 1.07
3435 reflections
60 parameters
H-atom parameters not refined
Δρ_max_ = 1.73 e Å^−3^
Δρ_min_ = −1.66 e Å^−3^
Absolute structure: Flack (1983 ▶), 1537 Friedel pairs
Flack parameter: −0.005 (6)

### 

Data collection: *APEX2* (Bruker, 2008 ▶); cell refinement: *SAINT* (Bruker, 2008 ▶); data reduction: *SAINT*; program(s) used to solve structure: *SHELXS97* (Sheldrick, 2008 ▶); program(s) used to refine structure: *SHELXL97* (Sheldrick, 2008 ▶); molecular graphics: *ATOMS for Windows* (Dowty, 2006 ▶); software used to prepare material for publication: *SHELXL97*.

## Supplementary Material

Crystal structure: contains datablocks I, global. DOI: 10.1107/S1600536809040355/fj2248sup1.cif

Structure factors: contains datablocks I. DOI: 10.1107/S1600536809040355/fj2248Isup2.hkl

Additional supplementary materials:  crystallographic information; 3D view; checkCIF report

## Figures and Tables

**Table:** 

Sr—O1^i^	2.6326 (10)
Sr—O2	2.6558 (10)
Sr—O3^ii^	2.6994 (10)
Na—O3^iii^	2.3926 (12)
Na—O2^iv^	2.4086 (12)

**Table:** 

O1⋯O5^v^	2.558 (7)
O1⋯O5	2.612 (8)
O1⋯O9^vi^	2.623 (8)
O1⋯O6^vii^	2.6640 (17)
O1⋯O4	2.7667 (14)
O1⋯O7	2.829 (5)
O1⋯O7^vi^	2.919 (5)
O2⋯O9^vi^	2.515 (9)
O2⋯O6^vii^	2.7488 (17)
O3⋯O8^vi^	2.562 (9)
O3⋯O6^vi^	2.6949 (17)
O3⋯O9^vi^	2.732 (9)
O3⋯O6	2.7492 (17)
O3⋯O5	2.757 (7)
O3⋯O7	2.776 (5)
O3⋯O7^vi^	2.930 (5)

Symmetry codes: (i) 
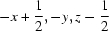
; (ii) 
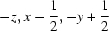
; (iii) 
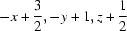
; (iv) 
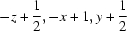
; (v) 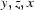; (vi) 
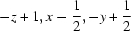
; (vii) 
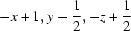
.

**Fig. 1.**
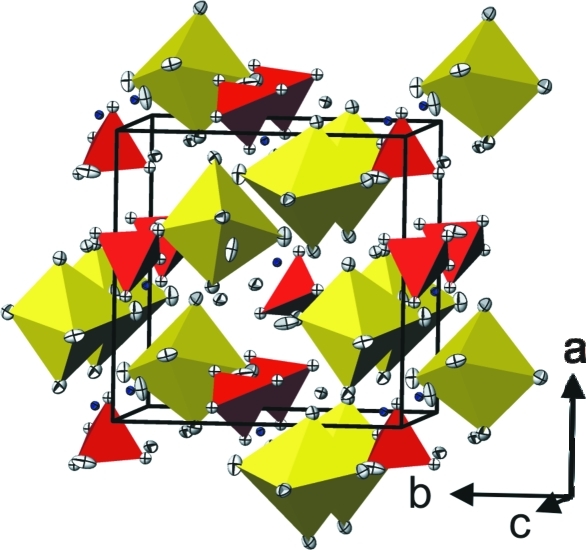
The crystal structure of NaSr(PO4)(H2O)9 in a projection approximately along [001]. Sr atoms are represented as blue spheres and O atoms as white spheres; NaO6 octahedra are given in yellow and AsO4 tetrahedra in red. For clarity, Sr—O bonds are omitted and only one orientation of the disordered AsO4 group is given. Ellipsoids are drawn at the 90% probability level.

## supplementary crystallographic information

### Comment

A previous study of NaSr(AsO_4_)(H_2_O)_9_ reports cubic symmetry and the lattice parameter as *a* = 10.70 Å (Ariguib-Kbir & Guerin, 1973). Moreover, isotypism with NaSr(PO_4_)(H_2_O)_9_ (Takagi *et al.*, 1982) was also revealed. Besides the synthetic phosphate analogue, NaSr(AsO_4_)(H_2_O)_9_ is also isotypic with the minerals nabaphite [NaBa(PO_4_)(H_2_O)_9_] (Baturin *et al.*, 1982) and nastrophite [Na(Sr,Ba)(PO_4_)(H_2_O)_9_] (Baturin *et al.*, 1981).

The crystal structures consist of slightly distorted Na(OH_2_)_6_ octahedra (3 symmetry) and *M*(OH_2_)_9_ tricapped trigonal prisms (3 symmetry), where *M* = Sr, Ba. These polyhedra share edges and establish a framework structure. Disordered *X*O_4_ tetrahedra (*X* = P, As; 3 symmetry) are situated in the interstitial space of the framework (Fig. 1).

The Na(H_2_O)_6_ octahedron is slightly distorted. The corresponding Na—O bond lenghts are in the usual range with an average of 2.40 Å, conform with the values in the isotypic compounds (NaSr(PO_4_)(H_2_O)_9_: 2.41 Å; nabaphite: 2.42 Å; nastrophite: 2.42 Å) and the sum of the ionic radii of 2.41 Å, as calculated for six-coordinated Na (Shannon, 1976).

The Sr^2+^ ion is surrounded by 9 oxygen atoms with an average Sr—O distance of 2.663 Å, in good agreement with the phosphate analogue (2.668 Å; Takagi *et al.*, 1982) and the sum of the ionic radii of 2.67 Å, as calculated for nine-coordinated Sr (Shannon, 1976).

The environment of the disordered AsO_4_ group consists of 16 water molecules with donor (D) — acceptor (A) distances between 2.5 and 3.0 Å (see Table 2). The overcrowding of water molecules (only 12 surrounding O atoms are expected, considering an ordered tetrahedral configuration for the arsenate unit with three donator atoms) may be the reason for the disorder of the AsO_4_ group. The average As—O bond length for the disordered AsO_4_ group is 1.685 Å, a value in very good agreement with those of 1.682 Å (Baur, 1981) and 1.686 Å (Schwendtner, 2008).

### Experimental

Crystals of the title compound were obtained during phase formation studies in the system Sr—As—O (Weil *et al.*, 2009) from hydrous solutions. All chemicals used were of analytical grade and employed without further purification. To a saturated Sr(OH)_2_^.^8H_2_O solution a diluted arsenic acid solution was dropwise added which resulted in a flocculent white precipitate (pH *ca*. 9). A concentrated NaOH solution was then added until a pH of 12 was reached. The resulting suspension was boiled for an hour. Then the precipitate was filtered off and the remaining solution was left to stand for several days. Besides few crystals of SrHAsO_4_ (Mihajlovic & Effenberger, 2006), colourless columnar crystals of the title compound up to several mm in length were obtained after complete evaporation of water.

### Refinement

The oxygen atoms O4 and O6 of the arsenate group were clearly discernible from Fourier maps. Consideration of full occupancy of these sites resulted in high electron densities of *ca* 4 e Å^3^ at a distance of *ca* 1.7 Å from As, indicating other disordered oxygen atoms. Therefore four additional O atoms were considered in the final model. Free refinement of the site occupancy factors (s.o.f.) of all six O atoms attached to arsenic resulted in a composition very close to the theoretical value. In the last refinement cycles the s.o.f.'s were constrained to meet the criterion for electroneutrality. The six disordered O atoms were finally refined with isotropic displacement parameters. No reasonable positions of the H atoms attached to the water molecules (O1, O2, O3) could be found in difference Fourier maps which may be due to the disorder of the AsO_4_ tetrahedron and the resulting complex hydrogen bonding scheme. Therefore all H atoms were excluded from the refinement. The highest remaining peak in the final difference Fourier map is 0.49 Å from As and the deepest hole is 0.43 Å from the same atom.

### Crystal data

**Table:**

NaSr(AsO_4_)(H_2_O)_9_	*D*_x_ = 2.268 Mg m^−^^3^
*M**_r_* = 411.67	Mo *K*α radiation, λ = 0.71073 Å
Cubic, *P*2_1_3	Cell parameters from 9782 reflections
Hall symbol: P 2ac 2ab 3	θ = 5.8–45.2°
*a* = 10.6435 (1) Å	µ = 7.29 mm^−^^1^
*V* = 1205.74 (2) Å^3^	*T* = 296 K
*Z* = 4	Fragment, colourless
*F*(000) = 816	0.36 × 0.24 × 0.24 mm

### Data collection

**Table:**

Bruker APEXII CCD diffractometer	3435 independent reflections
Radiation source: fine-focus sealed tube	3272 reflections with *I* > 2σ(*I*)
graphite	*R*_int_ = 0.037
ω and φ scans	θ_max_ = 45.7°, θ_min_ = 5.4°
Absorption correction: multi-scan (*SADABS*; Bruker, 2008)	*h* = −21→21
*T*_min_ = 0.179, *T*_max_ = 0.274	*k* = −20→21
41355 measured reflections	*l* = −17→17

### Refinement

**Table:**

Refinement on *F*^2^	H-atom parameters not refined
Least-squares matrix: full	*w* = 1/[σ^2^(*F*_o_^2^) + (0.0274*P*)^2^ + 0.5628*P*] where *P* = (*F*_o_^2^ + 2*F*_c_^2^)/3
*R*[*F*^2^ > 2σ(*F*^2^)] = 0.023	(Δ/σ)_max_ = 0.001
*wR*(*F*^2^) = 0.056	Δρ_max_ = 1.73 e Å^−^^3^
*S* = 1.07	Δρ_min_ = −1.66 e Å^−^^3^
3435 reflections	Extinction correction: *SHELXL97* (Sheldrick, 2008), Fc^*^=kFc[1+0.001xFc^2^λ^3^/sin(2θ)]^-1/4^
60 parameters	Extinction coefficient: 0.0147 (10)
0 restraints	Absolute structure: Flack (1983), 1537 Friedel pairs
Primary atom site location: structure-invariant direct methods	Flack parameter: −0.005 (6)
Secondary atom site location: difference Fourier map	

### Special details

**Table:**

Geometry. All e.s.d.'s (except the e.s.d. in the dihedral angle between two l.s. planes) are estimated using the full covariance matrix. The cell e.s.d.'s are taken into account individually in the estimation of e.s.d.'s in distances, angles and torsion angles; correlations between e.s.d.'s in cell parameters are only used when they are defined by crystal symmetry. An approximate (isotropic) treatment of cell e.s.d.'s is used for estimating e.s.d.'s involving l.s. planes.
Refinement. Refinement of *F*^2^ against ALL reflections. The weighted *R*-factor *wR* and goodness of fit *S* are based on *F*^2^, conventional *R*-factors *R* are based on *F*, with *F* set to zero for negative *F*^2^. The threshold expression of *F*^2^ > σ(*F*^2^) is used only for calculating *R*-factors(gt) *etc*. and is not relevant to the choice of reflections for refinement. *R*-factors based on *F*^2^ are statistically about twice as large as those based on *F*, and *R*- factors based on ALL data will be even larger.

### Fractional atomic coordinates and isotropic or equivalent isotropic displacement parameters (Å^2^)

**Table:**

	*x*	*y*	*z*	*U*_iso_*/*U*_eq_	Occ. (<1)
Sr	0.055001 (8)	0.055001 (8)	0.055001 (8)	0.00459 (3)	
As	0.421839 (11)	0.421839 (11)	0.421839 (11)	0.00709 (4)	
Na	0.67077 (6)	0.67077 (6)	0.67077 (6)	0.01193 (15)	
O1	0.39368 (12)	0.07909 (10)	0.35359 (10)	0.01687 (18)	
O2	0.29270 (9)	0.13067 (11)	0.04892 (10)	0.01519 (16)	
O3	0.60854 (10)	0.29633 (9)	0.14338 (10)	0.01243 (14)	
O4	0.33000 (13)	0.33000 (13)	0.33000 (13)	0.0077 (3)*	0.60
O5	0.4046 (7)	0.3186 (7)	0.3016 (7)	0.0117 (10)*	0.13
O6	0.56151 (12)	0.44234 (13)	0.35120 (12)	0.00759 (17)*	0.60
O7	0.5256 (5)	0.3069 (5)	0.3904 (5)	0.0115 (7)*	0.20
O8	0.5623 (8)	0.3833 (8)	0.4407 (8)	0.0088 (11)*	0.10
O9	0.5627 (8)	0.3668 (8)	0.4973 (8)	0.0084 (11)*	0.10

### Atomic displacement parameters (Å^2^)

**Table:**

	*U*^11^	*U*^22^	*U*^33^	*U*^12^	*U*^13^	*U*^23^
Sr	0.00459 (3)	0.00459 (3)	0.00459 (3)	0.00016 (2)	0.00016 (2)	0.00016 (2)
As	0.00709 (4)	0.00709 (4)	0.00709 (4)	−0.00024 (3)	−0.00024 (3)	−0.00024 (3)
Na	0.01193 (15)	0.01193 (15)	0.01193 (15)	0.00126 (17)	0.00126 (17)	0.00126 (17)
O1	0.0319 (5)	0.0088 (3)	0.0098 (3)	0.0043 (3)	−0.0045 (3)	−0.0005 (3)
O2	0.0109 (3)	0.0229 (4)	0.0118 (3)	−0.0039 (3)	0.0002 (3)	0.0007 (3)
O3	0.0130 (3)	0.0111 (3)	0.0132 (3)	0.0024 (3)	−0.0005 (3)	0.0015 (3)

### Geometric parameters (Å, °)

**Table:**

Sr—O1^i^	2.6326 (10)	As—O9^iv^	1.799 (9)
Sr—O1^ii^	2.6326 (10)	As—O9^v^	1.799 (9)
Sr—O1^iii^	2.6326 (10)	Na—O3^ix^	2.3926 (12)
Sr—O2^iv^	2.6558 (10)	Na—O3^x^	2.3926 (12)
Sr—O2	2.6558 (10)	Na—O3^xi^	2.3926 (12)
Sr—O2^v^	2.6558 (10)	Na—O2^xii^	2.4086 (12)
Sr—O3^vi^	2.6994 (10)	Na—O2^xiii^	2.4086 (12)
Sr—O3^vii^	2.6994 (10)	Na—O2^xiv^	2.4086 (12)
Sr—O3^viii^	2.6994 (10)	O4—O5^iv^	0.858 (7)
As—O8^iv^	1.563 (8)	O4—O5	0.858 (7)
As—O8	1.563 (8)	O4—O5^v^	0.858 (7)
As—O8^v^	1.563 (8)	O5—O5^v^	1.440 (13)
As—O6	1.6801 (13)	O5—O5^iv^	1.440 (13)
As—O6^v^	1.6801 (13)	O5—O7	1.602 (9)
As—O6^iv^	1.6801 (13)	O6—O8	1.141 (9)
As—O7^iv^	1.682 (5)	O6—O9^v^	1.462 (9)
As—O7	1.682 (5)	O6—O7	1.549 (5)
As—O7^v^	1.682 (5)	O6—O9	1.750 (8)
As—O4	1.693 (2)	O7—O8	1.049 (10)
As—O5^iv^	1.697 (7)	O7—O9	1.362 (9)
As—O5	1.697 (7)	O8—O9	0.627 (11)
As—O5^v^	1.697 (7)	O9—O6^iv^	1.462 (9)
As—O9	1.799 (9)		
			
O1···O5^iv^	2.558 (7)	O2···O6^xvi^	2.7488 (17)
O1···O5	2.612 (8)	O3···O8^xv^	2.562 (9)
O1···O9^xv^	2.623 (8)	O3···O6^xv^	2.6949 (17)
O1···O6^xvi^	2.6640 (17)	O3···O9^xv^	2.732 (9)
O1···O4	2.7667 (14)	O3···O6	2.7492 (17)
O1···O7	2.829 (5)	O3···O5	2.757 (7)
O1···O7^xv^	2.919 (5)	O3···O7	2.776 (5)
O2···O9^xv^	2.515 (9)	O3···O7^xv^	2.930 (5)
			
O1^i^—Sr—O1^ii^	80.79 (4)	O8^v^—As—O5^iv^	140.9 (4)
O1^i^—Sr—O1^iii^	80.79 (4)	O6—As—O5^iv^	128.5 (3)
O1^ii^—Sr—O1^iii^	80.79 (4)	O6^v^—As—O5^iv^	113.1 (3)
O1^i^—Sr—O2^iv^	86.96 (4)	O6^iv^—As—O5^iv^	80.9 (3)
O1^ii^—Sr—O2^iv^	134.39 (3)	O7^iv^—As—O5^iv^	56.6 (3)
O1^iii^—Sr—O2^iv^	140.21 (3)	O7—As—O5^iv^	81.6 (3)
O1^i^—Sr—O2	140.21 (3)	O7^v^—As—O5^iv^	106.7 (3)
O1^ii^—Sr—O2	86.96 (4)	O8^iv^—As—O5	140.9 (4)
O1^iii^—Sr—O2	134.39 (3)	O8—As—O5	91.7 (4)
O2^iv^—Sr—O2	75.03 (4)	O8^v^—As—O5	115.8 (4)
O1^i^—Sr—O2^v^	134.39 (3)	O6—As—O5	80.9 (3)
O1^ii^—Sr—O2^v^	140.21 (3)	O6^v^—As—O5	128.5 (3)
O1^iii^—Sr—O2^v^	86.96 (4)	O6^iv^—As—O5	113.1 (3)
O2^iv^—Sr—O2^v^	75.03 (4)	O7^iv^—As—O5	106.7 (3)
O2—Sr—O2^v^	75.03 (4)	O7—As—O5	56.6 (3)
O1^i^—Sr—O3^vi^	68.72 (3)	O7^v^—As—O5	81.6 (3)
O1^ii^—Sr—O3^vi^	68.05 (3)	O5^iv^—As—O5	50.2 (4)
O1^iii^—Sr—O3^vi^	138.94 (4)	O8^iv^—As—O9	80.4 (4)
O2^iv^—Sr—O3^vi^	66.55 (3)	O8^v^—As—O9	108.7 (4)
O2—Sr—O3^vi^	71.58 (3)	O8—As—O9	19.9 (4)
O2^v^—Sr—O3^vi^	134.06 (3)	O6^v^—As—O9	127.1 (3)
O1^i^—Sr—O3^vii^	68.05 (3)	O6—As—O9	60.3 (3)
O1^ii^—Sr—O3^vii^	138.94 (4)	O6^iv^—As—O9	49.6 (3)
O1^iii^—Sr—O3^vii^	68.72 (3)	O7^iv^—As—O9	104.4 (3)
O2^iv^—Sr—O3^vii^	71.58 (3)	O7—As—O9	45.9 (3)
O2—Sr—O3^vii^	134.06 (3)	O7^v^—As—O9	134.8 (3)
O2^v^—Sr—O3^vii^	66.55 (3)	O4—As—O9	123.4 (3)
O3^vi^—Sr—O3^vii^	119.992 (1)	O5^iv^—As—O9	110.0 (4)
O1^i^—Sr—O3^viii^	138.94 (4)	O5—As—O9	102.5 (4)
O1^ii^—Sr—O3^viii^	68.72 (3)	O5^v^—As—O9	152.0 (4)
O1^iii^—Sr—O3^viii^	68.05 (3)	O8^iv^—As—O9^iv^	19.9 (4)
O2^iv^—Sr—O3^viii^	134.06 (3)	O8^v^—As—O9^iv^	80.4 (4)
O2—Sr—O3^viii^	66.55 (3)	O8—As—O9^iv^	108.7 (4)
O2^v^—Sr—O3^viii^	71.58 (3)	O6^v^—As—O9^iv^	49.6 (3)
O3^vi^—Sr—O3^viii^	119.992 (1)	O6—As—O9^iv^	127.1 (3)
O3^vii^—Sr—O3^viii^	119.992 (1)	O6^iv^—As—O9^iv^	60.3 (3)
O8^iv^—As—O8	99.3 (4)	O7^iv^—As—O9^iv^	45.9 (3)
O8^iv^—As—O8^v^	99.3 (4)	O7—As—O9^iv^	134.8 (3)
O8—As—O8^v^	99.3 (4)	O7^v^—As—O9^iv^	104.4 (3)
O8^iv^—As—O6	130.1 (3)	O4—As—O9^iv^	123.4 (3)
O8^v^—As—O6	69.2 (3)	O5^iv^—As—O9^iv^	102.5 (4)
O8^iv^—As—O6^v^	69.2 (3)	O5—As—O9^iv^	152.0 (4)
O8—As—O6^v^	130.1 (3)	O5^v^—As—O9^iv^	110.0 (4)
O6—As—O6^v^	109.83 (4)	O9—As—O9^iv^	92.6 (4)
O8—As—O6^iv^	69.2 (3)	O8^iv^—As—O9^v^	108.7 (4)
O8^v^—As—O6^iv^	130.1 (3)	O8^v^—As—O9^v^	19.9 (4)
O6—As—O6^iv^	109.83 (4)	O8—As—O9^v^	80.4 (4)
O6^v^—As—O6^iv^	109.83 (4)	O6^v^—As—O9^v^	60.3 (3)
O8—As—O7^iv^	124.0 (4)	O6—As—O9^v^	49.6 (3)
O8^v^—As—O7^iv^	117.1 (3)	O6^iv^—As—O9^v^	127.1 (3)
O6—As—O7^iv^	164.45 (18)	O7^iv^—As—O9^v^	134.8 (3)
O6^v^—As—O7^iv^	76.41 (17)	O7—As—O9^v^	104.4 (3)
O6^iv^—As—O7^iv^	54.87 (18)	O7^v^—As—O9^v^	45.9 (3)
O8^iv^—As—O7	117.1 (3)	O4—As—O9^v^	123.4 (3)
O8^v^—As—O7	124.0 (4)	O5^iv^—As—O9^v^	152.0 (4)
O6—As—O7	54.87 (18)	O5—As—O9^v^	110.0 (4)
O6^v^—As—O7	164.45 (18)	O5^v^—As—O9^v^	102.5 (4)
O6^iv^—As—O7	76.41 (17)	O9—As—O9^v^	92.6 (4)
O7^iv^—As—O7	117.62 (9)	O9^iv^—As—O9^v^	92.6 (4)
O8^iv^—As—O7^v^	124.0 (4)	O3^ix^—Na—O3^x^	89.64 (5)
O8—As—O7^v^	117.1 (3)	O3^ix^—Na—O3^xi^	89.65 (5)
O6—As—O7^v^	76.41 (17)	O3^x^—Na—O3^xi^	89.64 (5)
O6^v^—As—O7^v^	54.87 (18)	O3^ix^—Na—O2^xii^	75.47 (4)
O6^iv^—As—O7^v^	164.45 (18)	O3^x^—Na—O2^xii^	155.21 (3)
O7^iv^—As—O7^v^	117.62 (9)	O3^xi^—Na—O2^xii^	109.62 (4)
O7—As—O7^v^	117.62 (9)	O3^ix^—Na—O2^xiii^	109.62 (4)
O8^iv^—As—O4	118.4 (3)	O3^x^—Na—O2^xiii^	75.47 (4)
O8—As—O4	118.4 (3)	O3^xi^—Na—O2^xiii^	155.21 (3)
O8^v^—As—O4	118.4 (3)	O2^xii^—Na—O2^xiii^	90.76 (5)
O6—As—O4	109.11 (5)	O3^ix^—Na—O2^xiv^	155.21 (3)
O6^v^—As—O4	109.11 (5)	O3^x^—Na—O2^xiv^	109.62 (4)
O6^iv^—As—O4	109.11 (5)	O3^xi^—Na—O2^xiv^	75.47 (4)
O7^iv^—As—O4	81.04 (17)	O2^xii^—Na—O2^xiv^	90.76 (5)
O7—As—O4	81.04 (17)	O2^xiii^—Na—O2^xiv^	90.76 (5)
O7^v^—As—O4	81.04 (17)	Na^xvi^—O2—Sr	103.37 (4)
O8^iv^—As—O5^iv^	91.7 (4)	Na^xvii^—O3—Sr^xviii^	102.52 (4)
O8—As—O5^iv^	115.8 (4)		

Symmetry codes: (i) −*y*, *z*−1/2, −*x*+1/2; (ii) −*x*+1/2, −*y*, *z*−1/2; (iii) *z*−1/2, −*x*+1/2, −*y*; (iv) *y*, *z*, *x*; (v) *z*, *x*, *y*; (vi) −*y*+1/2, −*z*, *x*−1/2; (vii) −*z*, *x*−1/2, −*y*+1/2; (viii) *x*−1/2, −*y*+1/2, −*z*; (ix) −*y*+1, *z*+1/2, −*x*+3/2; (x) *z*+1/2, −*x*+3/2, −*y*+1; (xi) −*x*+3/2, −*y*+1, *z*+1/2; (xii) *y*+1/2, −*z*+1/2, −*x*+1; (xiii) −*z*+1/2, −*x*+1, *y*+1/2; (xiv) −*x*+1, *y*+1/2, −*z*+1/2; (xv) −*z*+1, *x*−1/2, −*y*+1/2; (xvi) −*x*+1, *y*−1/2, −*z*+1/2; (xvii) −*x*+3/2, −*y*+1, *z*−1/2; (xviii) *x*+1/2, −*y*+1/2, −*z*.
